# Supplementary material for: Tuning the performance of a micrometer-sized Stirling engine through reservoir engineering
Source: Nat Commun. 2021 Aug 13;12:4927. doi: 10.1038/s41467-021-25230-1 (PMC8363610; doi:10.1038/s41467-021-25230-1)
Supplement: Supplementary file 1 — Supplementary Information [file 41467_2021_25230_MOESM1_ESM.pdf]

Supplementary Information for

Tuning the performance of a micrometer-sized Stirling engine through reservoir engineering

Niloyendu Roy,[1] * Nathan Leroux,[2] A K Sood,[3,4] and Rajesh Ganapathy[4,5]

[1]*Chemistry and Physics of Materials Unit,*

Jawaharlal Nehru Centre for Advanced Scientific Research,

Jakkur, Bangalore - 560064, INDIA

[2]*Unité Mixte de Physique CNRS/Thales, 91767 Palaiseau, France*

[3]*Department of Physics, Indian Institute of Science, Bangalore- 560012, INDIA*

[4]*International Centre for Materials Science,*

Jawaharlal Nehru Centre for Advanced Scientific Research,

Jakkur, Bangalore - 560064, INDIA

[5]*School of Advanced Materials (SAMat),*

Jawaharlal Nehru Centre for Advanced Scientific Research,

Jakkur, Bangalore - 560064, INDIA

* Corresponding author. email: niloycrj@gmail.com

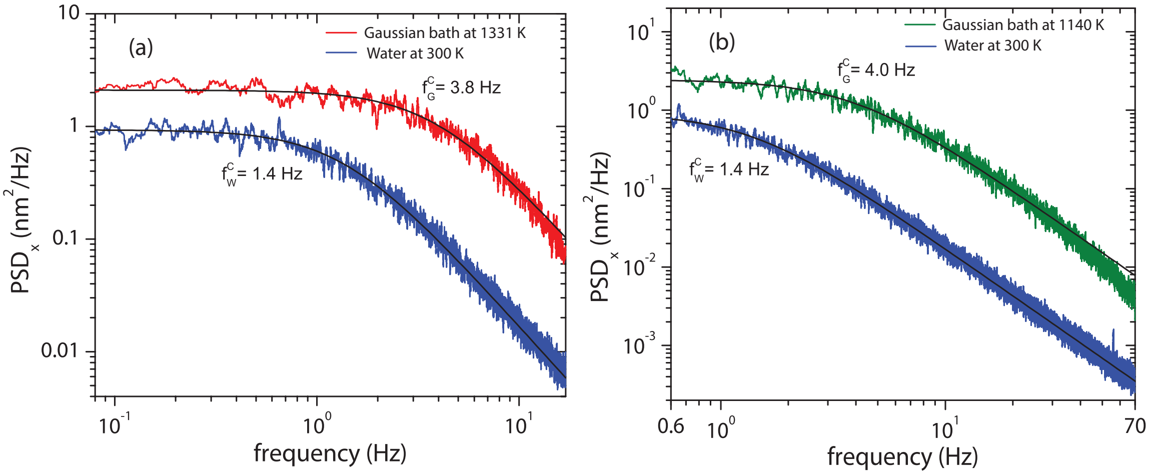

Supplementary Figure 1: Power spectral density (PSD) in Gaussian reservoirs. (a) PSD of the position x of the particle trapped in pure water is showed by the blue line with lower saturation value. PSD of the particle's position x in the engineered Gaussian bath at 1331 K is represented by the red line with higher saturation value. This bath was created by flashing the secondary trap at 34 Hz. Black lines are fits to Supplementary eq. (1) overlaid on the experimental data. Fits result in a roll-off frequency of $f_w^c = 1.4$ Hz for the PSD in pure water and a roll-off requency of $f_{\rm G}^c = 3.86$ Hz for the PSD in the engineered Gaussian bath. **b** PSD of the position x of the particle trapped in pure water is showed by the blue line with lower saturation value. PSD of the particle's position x in the engineered Gaussian bath at 1140 K is represented by the green line with higher saturation value. This bath was created by flashing the secondary trap at 135 Hz. Black lines are fits to Supplementary eq. (1) resulting in a roll-off frequency of $f_w^c = 1.4$ Hz for the PSD in pure water and a roll-off frequency of $f_{\rm G}^c = 4.0$ Hz in the engineered Gaussian reservoir at 1140 K.

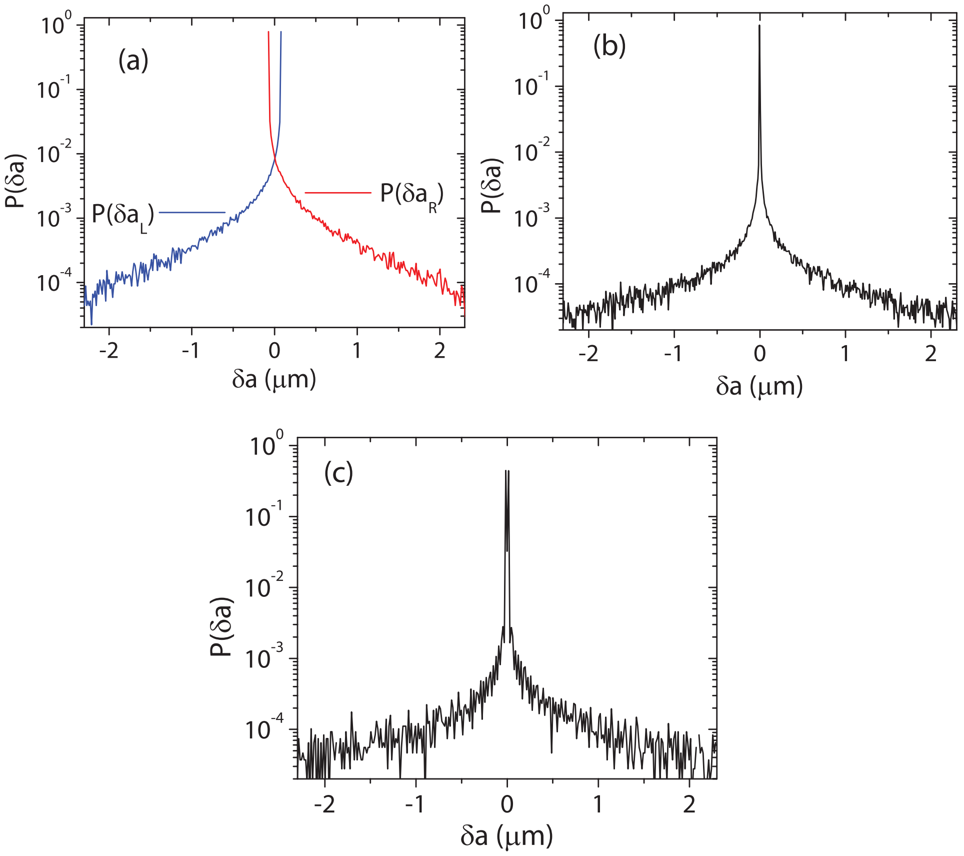

Supplementary Figure 2: δa distribution for non-Gaussian reservoir engineering. In **(a)** skewed distributions generated for the construction of δa is plotted. Blue line shows the negatively skewed distribution δa_{L}. Red line shows the positively skewed distribution δa_{R}. **(b)** shows the δa distribution used to engineer a non-Gaussian reservoir with $\kappa = 20$ at 1824 K. To create this reservoir, the secondary trap was flashed at 34 Hz. **(c)** shows the δa distribution used to engineer a non-Gaussian reservoir with $\kappa = 10$ at 1500 K. To create this reservoir, the secondary trap was flashed at 135 Hz.

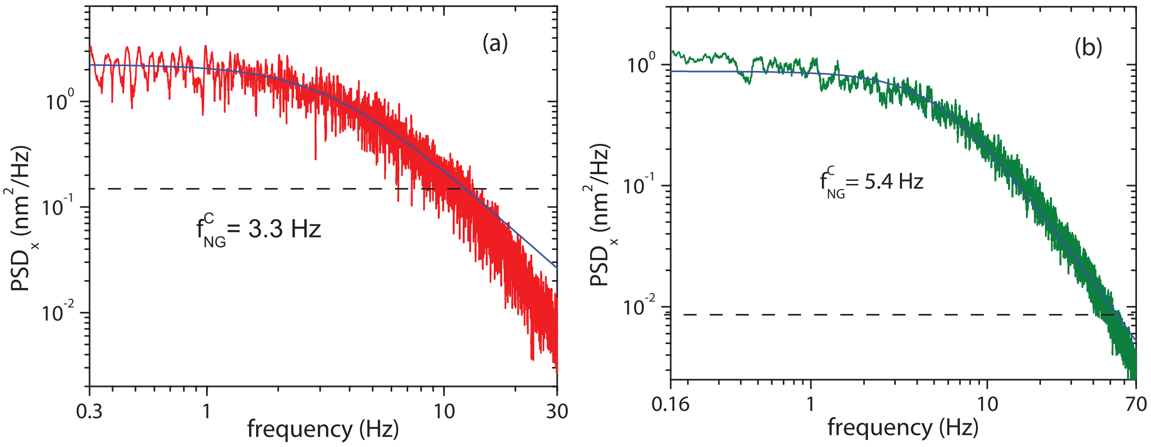

Supplementary Figure 3: Power spectral density (PSD) in non-Gaussian reservoirs (a) PSD of the position x of the particle in the non-Gaussian reservoir with $\kappa = 20$ at 1310 K is showed (red line). This reservoir was created by flashing the secondary trap at 34 Hz. Blue line is a fit to Supplementary eq. (1) resulting in a roll-off frequency $f^c_{\mathrm{NG}} = 3.98$ Hz. We see deviations from the fit after about one order-of-magnitude fall (dashed line). **(b)** PSD of the position x of the particle in the non-Gaussian reservoir with $\kappa = 10$ at 1500 K is showed (green line). This reservoir was created by flashing the secondary trap at 135 Hz. Blue line is a fit to Supplementary eq. (1) resulting in a roll-off frequency $f^c_{\mathrm{NG}} = 5.4$ Hz. Here the fit agrees with the data over a broader dynamic range (dashed line).

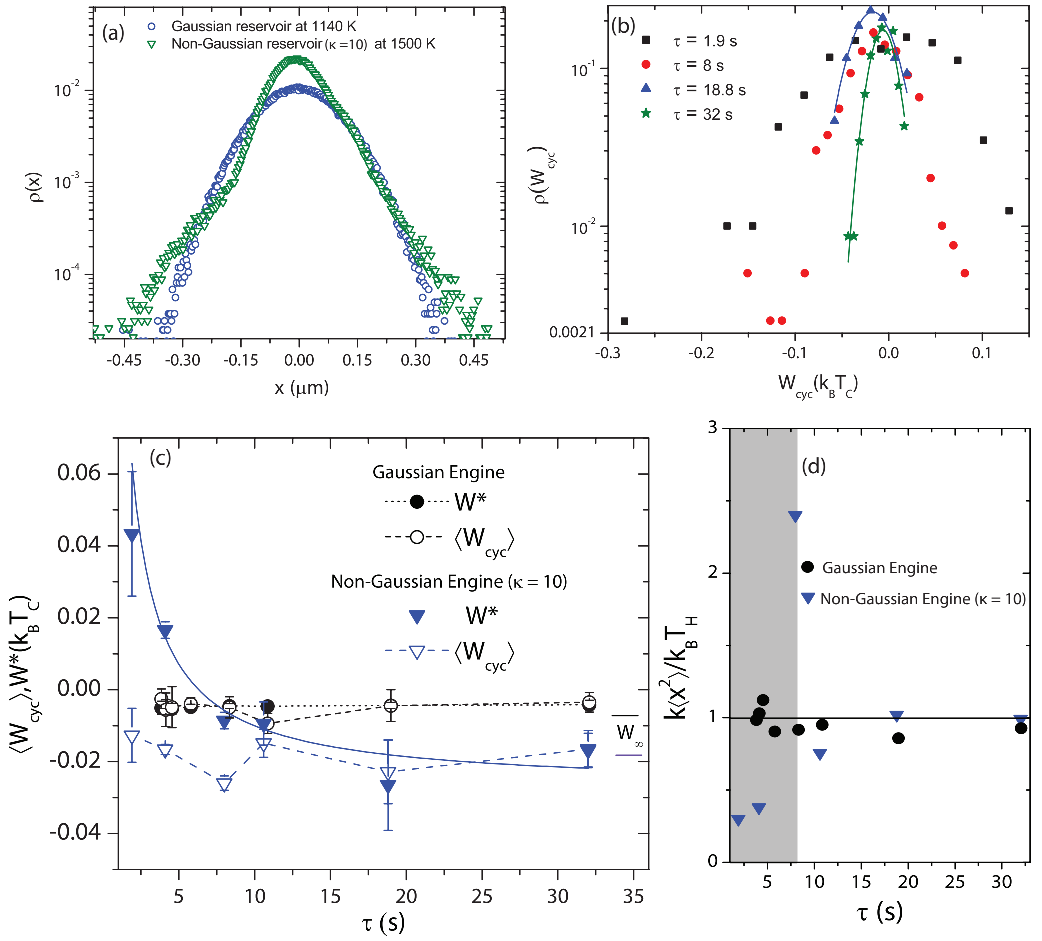

Supplementary Figure 4: Probability distribution of position, work and origins of irreversibility in the non-Gaussian engine with $\kappa = 10$ at the hot reservoir . (a) Equilibrium probability distribution $\rho(x)$ of the particle's position in the cold Gaussian reservoir at 1140 K is showed by the blue hollow circles and in the hot non-Gaussian reservoir with $\kappa = 10$ at 1500 K is showed by the green hollow triangles. In **(b)**, probability distribution of work done per cycle, $\rho(W_{\text{cyc}})$, for the non-Gaussian engine with $\kappa = 10$ at the hot reservoir for various τ. **(c)** Blue hollow and solid triangles show the average work done per cycle $\langle W_{\text{cyc}} \rangle$ and the most-probable work W^*, respectively, for the non-Gaussian engine with $\kappa = 10$ for the hot reservoir at various τ. The blue solid line is a fit to eq. (1) of the main text. Black hollow and solid circles show $\langle W_{\text{cyc}} \rangle$ and W^*, respectively, for the thermal/Gaussian engine. The experimentally calculated work for these engines agree with the theoretically calculated quasistatic work W_∞ at large τ. The value of W_∞ is shown by the blue short horizontal line for the non-Gaussian engine and by the black line for the Gaussian engine. Mean work $\langle W_{\text{cyc}} \rangle$ is calculated for each realization of the engine over these many cycles: τ = 1.9 s (225 cycles), τ = 4 s (200 cycles), τ = 8 s (100 cycles), τ = 10.6 s (100 cycles), τ = 18.8 s (50 cycles) and τ = 32 s (25 cycles) for the non-Gaussian engine. **(d)** The ratio measuring equilibrium violation, $k\langle x^2 \rangle / k_{\text{B}} T^{\text{H}}_{\text{eff}}$, calculated at the midpoint of the hot isotherm for different τ is showed by the blue triangles for the non-Gaussian engine with $\kappa = 10$ in the hot reservoir and by the black circles for the Gaussian engine. The horizontal line indicates the equilibrium condition which is violated inside the shaded grey region, in case of the non-Gaussian engine with $\kappa = 10$ in the hot reservoir. The error bars indicate the standard deviations of the mean and the most probable quantities across different experiments.

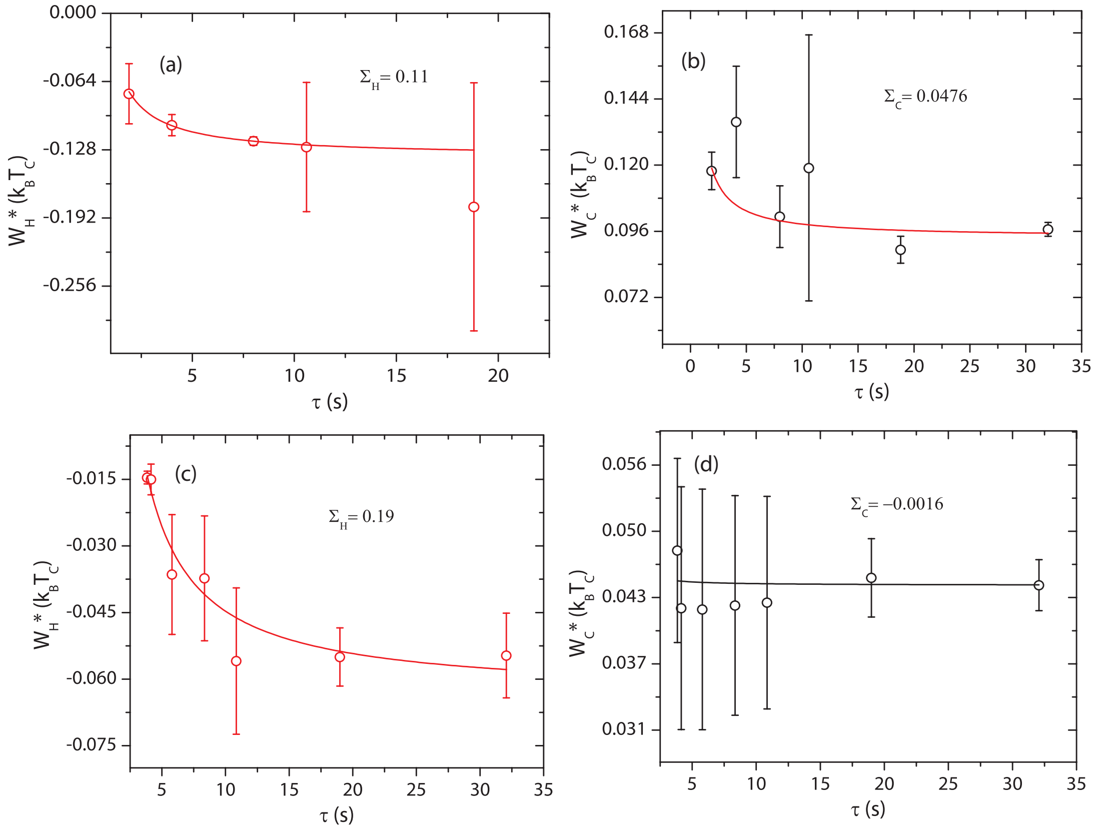

Supplementary Figure 5: Work done per isotherm for the non-Gaussian engines. **(a)** Red circles denote the most probable work $W_{\rm H}^*$ performed during the hot isotherm as a function of cycle time τ for the non-Gaussian engine with $\kappa = 10$ at the hot reservoir. The red line indicates a fit to Supplementary eq. (6) yielding $\Sigma_{\rm H} = 0.11$. **(b)** Black circles denote the most probable work $W_{\rm C}^*$ performed during the cold isotherm for the non-Gaussian engine with $\kappa = 10$ at the hot reservoir. The black line is a fit to Supplementary eq. (7) resulting in $\Sigma_{\rm C} = 0.0476$. **(c)** Red circles denote the most probable work $W_{\rm H}^*$ performed during the hot isotherm as a function of cycle time τ for the non-Gaussian engine with $\kappa = 20$ at the hot reservoir. The red line indicates a fit to Supplementary eq. (6) yielding $\Sigma_{\rm H} = 0.19$. **(d)** Black circles denote the most probable work $W_{\rm C}^*$ performed during the cold isotherm for the non-Gaussian engine with $\kappa = 20$ at the hot reservoir. The black line is a fit to Supplementary eq. (7) resulting in $\Sigma_C = -0.0016 \sim 0$. The error bars indicate the standard deviations of the most probable across different experiments.

Supplementary Note 1 | Determination of secondary trap's stiffness and effective temperature of the engineered reservoirs

Power spectral density (PSD) of the particle's position is given by the Fourier transform of its auto-correlation function. When trapped in pure water, PSD as a function of frequency (f) shows a Lorentzian profile [1] given by

$$\mathrm{PSD}(f) = \frac{1}{2\pi^2\gamma}\frac{k_{\rm B}T}{f^2 + f_w^{c2}} \tag{1}$$

where γ is the damping coefficient, $f_w^c = k_1/(2\pi\gamma)$ is the roll-off frequency proportional to the primary trapping stiffness k_1 and T is the temperature of water that is maintained at 300 K in all our experiments. In presence of a flashing secondary trap (of stiffness k_2) with memoryless Gaussian fluctuations, the overall PSD must remain a Lorentzian with a roll-off frequency f_G^c proportional to the effective stiffness $k = k_1 + k_2$ and an effective temperature that is higher than that of the bath. In Supplementary Figure 1a, PSD in the absence of external noise (blue line) fits to eq. (1) with roll-off frequency $f_w^c = 1.35$ Hz which corresponds to a primary trapping stiffness $k_1 = 0.702$ pN μm^{-1} measured using equipartition relation. With external Gaussian noise at 34 Hz imposed, represented in Fig 1b of the main text, Lorentzian fit yields $f_{\mathrm{G}}^c = 3.86$ Hz, allowing us to calculate $k = \frac{f_{\mathrm{G}}^c}{f_w^c} k_1 \equiv 2$ pNμm^{-1} and thus $k_2 = k - k_1 \equiv 1.29$ pNμm^{-1}. Using this value of k in equipartition ($k\langle x^2\rangle = k_{\mathrm{B}}T_{\mathrm{eff}}$), the effective temperature of the engineered Gaussian reservoir is finally evaluated to be $T_{\mathrm{eff}} = 1331$ K. A higher T_{eff} lifts the plateau of the Lorentzian profile as shown in Supplementary Figure 1. Supplementary Figure 1b shows the corresponding PSDs with noise imposed at 135 Hz. The above mentioned calculation yields $k_1 = 0.5$ pN μm^{-1}, $k_2 = 3.17$ pN μm^{-1} and $k = 3.67$ pN μm^{-1}. Note that, since the residence time at each δa position is reduced due to the high flashing frequency, the value of k_2 is higher in this case.

Due to the memoryless nature of the δa-distribution, no further correlation or drag is added to the particle dynamics even in the engineered non-Gaussian reservoir. Therefore the overall PSD should still be a Lorentzian which is confirmed in Supplementary Figure 3. The black line indicates the fit (Supplementary Figure 3a) to Supplementary eq. (1) and the roll-off frequency $f_{\mathrm{NG}}^c = 3.3$ Hz seems to match f_{G}^c for the non-Gaussian reservoir with $\kappa = 27$, created by flashing the secondary trap at 34 Hz. This implies an effective stiffness of $k = 2.06$ pNμm^{-1} and an effective temperature $T_{\mathrm{eff}} = 1310$ k corresponding to the engineered non-Gaussian noise that is represented in Figure 1c of the main text.We see that for this reservoir, the PSD fits to a Lorentzian up to 20 Hz and then decays down. This is a result of low flashing frequency (34 Hz). For the non-Gaussian reservoir with $\kappa = 10$ at the hot reservoir which is created by flashing the secondary trap at 135 Hz, the PSD (Supplementary Figure 3b) fits to a lorentzian for almost two decades, even strengthening the claim of memoryless nature of our noise. We performed two sets of experiments of Stirling engine. One, with flashing the secondary trap at 34 Hz and the other with flashing the secondary trap at 135 Hz. Work done and efficiency of both these engines agreed with the theoretically calculated quasistatic work and Stirling saturation efficiency. Therefore, the disagrrement of the fit on Supplementary Figure 3a beyond 20 Hz does not seems to affect the physics of our engines and our engineered noise is effectively additive and uncorrelated.

Supplementary Note 2 | Stirling engine with engineered Gaussian baths

The performance of our non-Gaussian engines were compared to a Stirling engine operating in the same range of τ as the non-Gaussian ones between two engineered Gaussian baths with effective temperatures $T_{\mathrm{eff}}^{\mathrm{H}} = 1378$ K and $T_{\mathrm{eff}}^{\mathrm{C}} = 1238$ K, resulting in a $\Delta T_{\mathrm{eff}} = 140$ K. The isotherms of this engine were performed between a maximum and minimum effective stiffness of $k_{\mathrm{max}} = 2.29$ pNμm^{-1} and $k_{\mathrm{min}} = 2.08$ pNμm^{-1}, respectively, resulting in an identical expansion/compression ratio as the non-Gaussian engines.

Supplementary Note 3 | Strength of noise-statistics tuning on modulating engine performance

A trade-off between increasing irreversibility and decreasing τ causes the power output of such a Stirling engine to go through a maximum and eventually a fall on lowering the cycle time. As a result of negligible irreversibility, the power output (Figure 3a of main text) of this Gaussian engine increases monotonically on lowering τ and the maximum is expected to occur at a much smaller cycle time which is not accessible in our experiments. We realize that when the compression/expansion ratio is held fixed, an elevation of $T_{\text{eff}}^{\text{H}}$ of the hot Gaussian bath facilitates volume equilibriation so that the onset of irreversibility occurs only for a even faster isothermal expansion. Therefore a hypothetical Gaussian engine (with identical conditions as the non-Gaussian ones) that performs the isothermal expansion at a considerably higher $T_{\text{eff}}^{\text{H}}$ ($= 1824$ K or 1540 K) than our experimental Gaussian engine, is expected to exhibit the power peak at a even smaller τ than the experimental one. This implies even a stronger effect of noise statistics-tuning on the power-peak modulation - than what one can immediately infer from Figure 3a of the main text.

Supplementary Note 4 | Non-Gaussian Stirling engine having a hot reservoir with $\kappa = 10$

To elucidate the effect of non-Gaussian statistics on the performance of a stochastic Stirling engine, we executed Stirling cycles at different operating speed between a cold Gaussian reservoir and a hot non-Gaussian reservoir with $\kappa = 20$ in one set of experiments and $\kappa = 10$ in the other. The engine protocol with $\kappa = 20$ at the hot reservoir is described in details in Figure 1e of the main text. The non-Gaussian engine with $\kappa = 10$ at the hot reservoir was executed following an identical protocol as the one described in Figure 1e. This engine was performed by linearly varying the effective stiffness of the confining potential between $k_{\text{max}} = 4.04$ pNμm^{-1} and $k_{\text{min}} = 3.67$ pNμm^{-1} implying an identical expansion/compression ratio as the one with $\kappa = 20$ at the hot reservoir. The effective temperatures of the hot and the cold reservoir between which this engine performed, were set to $T_{\text{eff}}^{\text{H}} = 1500$ k and $T_{\text{eff}}^{\text{C}} = 1140$ k, respectively, so that $\Delta T_{\text{eff}} = 360$ K is comparable to that of the non-Gaussian engine with $\kappa = 20$ at the hot reservoir. Supplementary Figure 4a shows the probability distribution $\rho(x)$ of the particle's position in the cold Gaussian and the hot non-Gaussian reservoir. It can be clearly observed that the central part of $\rho(x)$ for the non-Gaussian reservoir with $\kappa = 10$, is broader than that of the corresponding $\rho(x)$ in reservoirs with $\kappa = 27$ (Figure 1d of the main text) or $\kappa = 20$ (Figure 1e of the main text), in spite of having comparatively lower T_{eff}. Supplementary Figure 4b shows the work distribution $\rho(W_{\text{cyc}})$ for $\tau = 18.8$ s, 8 s and 1.9 s. We see that the increase in the negative skewness of $\rho(W_{\text{cyc}})$ with lowering τ is less compared to the non-Gaussian engine with $\kappa = 20$ at the hot reservoir as described in Figure 2b of the main text. This can be explained by the fact that a decrease in the kurtosis of $\rho(x)$ decreases the magnitude and the number of displacement spikes encountered within any given time interval, which, in turn, reduces the possibility of anomalously large negative work in few cycles. In Supplementary Figure 4c, W^* of the non-Gaussian engine remains negative till $\tau = 8$ s and turns positive indicating high irreversibility for $\tau \leq 6$ s. The sole effect of non-Gaussian statistics of the hot reservoir in this irreversibility-build-up is again guaranteed by the fact that the Gaussian engine has a lower

effective temperature of the hot reservoir ($T^{\mathrm{H}}_{\mathrm{eff}} = 1378$ K) and yet shows an exhaustive volume equilibriation for all τ in most of the cycles (since W^* has a flat profile). This build-up of irreversibility resulting in W^* lift-off for the non-Gaussian engine with $\kappa = 10$ at the hot reservoir is consistent with equilibrium violation, which is confirmed by the deviation of $k\langle x^2\rangle/k_{\mathrm{B}}T_{\mathrm{H}}$ from the value 1 (equipartition) below $\tau \leq 8$ s (Supplementary Figure 4d). More interestingly, a comparison of Supplementary Figure 4c and Figure 2c of the main text firmly implies that the onset of irreversibility for this engine occurs at a τ smaller than the one with $\kappa = 20$ at the hot reservoir. This is a result of the reduced kurtosis of $\rho(x)$ with a fatter central portion which causes a higher possibility of exploration of the permitted volume in most of the cycles during the isothermal expansion steps. Therefore, indeed the kurtosis of the hot reservoir plays a pivotal role in deciding the onset of irreversibility when a non-Gaussian engine is operated at finite cycle times.

Supplementary Note 5 | Calculation of most probable efficiency

The efficiency of the engine is given by the ratio of work done per cycle to the heat absorbed from the hot bath, that is $\varepsilon = \frac{W_{\mathrm{cyc}}}{Q}$. To capture the effect of noise-induced irreversibility we use the most probable work done per cycle W^* in this calculation. The total heat Q absorbed from the hot reservoir consists of the heat $Q_{2\to3}$ (eq. (2) of the main text) taken from the hot bath while the particle is decoupled and coupled from the cold to the hot bath during the isochoric transition ②→③ and the heat $Q_{3\to4}$ (eq. (3) of the main text) dissipated during the isothermal transition ③→④. Since the isochoric transition ②→③ occurs instantaneously in our engine, we directly calculate it from the expected change in internal energy of the system before and after the transition as $Q_{2\to3} = -\frac{1}{2}k_{\mathrm{B}}(T^{\mathrm{H}}_{\mathrm{eff}} - T^{C}_{\mathrm{eff}}) \equiv \langle Q_{\mathrm{isochoric}}\rangle$. The heat dissipated during the isothermal expansion is given by

$$Q_{3\to4} = \int_{(3)}^{(4)} \frac{\partial U}{\partial x}\dot{x}dt \equiv \int_{(3)}^{(4)} kx\dot{x}dt \tag{2}$$

Integration by parts of the right most expression in Supplementary eq. (2) yields

$$Q_{3\to4} = \frac{1}{2}[k(t)x(t)^2]^{(4)}_{(3)} - \frac{1}{2}\int_{(3)}^{(4)} x^2 \circ dk. \tag{3}$$

The first term in the right hand side of Supplementary eq. (3) is called the boundary term (Q_{boundary}) calculated as

$$Q_{\mathrm{boundary}} = k_{\mathrm{min}}\langle x^2\rangle_{(4)} - k_{\mathrm{max}}\langle x^2\rangle_{(3)}. \tag{4}$$

In our experiments Q_{boundary} shows a symmetric distribution centred around zero and hence we take the average of this quantity for efficiency calculation. The second term is the work W_{H} performed during the isothermal expansion in the hot reservoir. Since this quantity has an asymmetric distribution even in case of a Gaussian heat bath, we use the average of this quantity. Therefore the most probable efficiency of the non-Gaussian engine is given by

$$\varepsilon^* = \frac{W^*}{\langle W_{\mathrm{H}}\rangle + \langle Q_{\mathrm{boundary}}\rangle + \langle Q_{\mathrm{isochoric}}\rangle}. \tag{5}$$

Supplementary Note 6 | Calculation of Curzon-Ahlborn efficiency

Work performed during the isothermal expansion (W_{H}) and the compression (W_{C}) can be written as [2]:

$$W_{\mathrm{H}} = W_{\mathrm{H}_\infty} + W_{\mathrm{H}_{\mathrm{irr}}} \equiv W_{\mathrm{H}_\infty} + \Sigma_{\mathrm{H}}/\tau \tag{6}$$

and

$$W_{\mathrm{C}} = W_{\mathrm{C}_\infty} - W_{\mathrm{C}_{\mathrm{irr}}} \equiv W_{\mathrm{C}_\infty} - \Sigma_{\mathrm{C}}/\tau \tag{7}$$

where W_{H_∞} and W_{C_∞} are corresponding quasistatic work. Fast driving causes an irreversible work $W_{\mathrm{H}_{\mathrm{irr}}}$ ($W_{\mathrm{C}_{\mathrm{irr}}}$) which grows inversely with τ with irreversibility parameters Σ_{H} (Σ_{C}). It has been shown by Schmiedl et al. [2] that the efficiency at maximum power of such a stochastic heat engine can be written in terms of the quasistatic saturation efficiency using the irreversibility parameters as:

$$\varepsilon_{\mathrm{CA}} = \frac{\varepsilon_{\mathrm{sat}}}{2 - \alpha\varepsilon_{\mathrm{sat}}} \tag{8}$$

where $\alpha = 1/(1 + \sqrt{\Sigma_{\mathrm{C}}/\Sigma_{\mathrm{H}}})$.

Although the above relations have been deduced for average quantities, we check its validity in the most probable case. Fitting W^* and W^*_{C} to Supplementary eq. (6) and (7) respectively , we find $\Sigma_{\mathrm{H}} = 0.11$ and $\Sigma_{\mathrm{C}} = 0.047$ (Supplementary Figure 5a and 5b $\alpha = 0.6$ for the non-Gaussian engine with $\kappa = 10$ at the hot reservoir and $\Sigma_{\mathrm{H}} = 0.19$ and $\Sigma_{\mathrm{C}} \sim 0$ (Supplementary Figure 5c and 5d) resulting in an $\alpha \sim 1$ for the non-Gaussian engine with $\kappa = 20$ at the hot reservoir. This yields an $\varepsilon_{\mathrm{CA}} = 0.035$ and $\varepsilon_{\mathrm{CA}} = 0.0256$ for the engines with $\kappa = 10$ and $\kappa = 20$ at the hot reservoirs respectively. Most interestingly, these values of $\varepsilon_{\mathrm{CA}}$ agree with $\varepsilon_{\mathrm{Max}} = 0.027$ ($\tau = 8$ s) for the engine with $\kappa = 10$ at the hot reservoir and $\varepsilon_{\mathrm{Max}} = 0.025$ ($\tau = 10.6$ s) for the engine with $\kappa = 20$ at the hot reservoir.

Supplementary References

[1] Martinez, I. A., Roldán, E., Parrondo, J. M. & Petrov, D. Effective heating to several thousand kelvins of an optically trapped sphere in a liquid. *Phys. Rev. E* **87**, 032159 (2013).

[2] Schmiedl, T. & Seifert, U. Efficiency at maximum power: An analytically solvable model for stochastic heat engines. *Euro. Phys. Lett.* **81**, 20003 (2007).
