# Supplementary material for: Tuning the performance of a micrometer-sized Stirling engine through reservoir engineering
Source: Nat Commun. 2021 Aug 13;12:4927. doi: 10.1038/s41467-021-25230-1 (PMC8363610; doi:10.1038/s41467-021-25230-1)
Supplement: Supplementary file 3 — Description of Additional Supplementary Files [file 41467_2021_25230_MOESM3_ESM.pdf]

Description of Additional Supplementary Files

File name: Supplementary Movie 1
Description: 4x slow motion video of the trapped colloidal particle in engineered non Gaussian reservoir is presented. This reservoir was created by flashing the secondary trap at a constant frequency of 34 Hz. Most of the time, the particle remains confined close to its time-averaged centre. Over the entire duration of 9.21 s, the particle undergoes large excursions only five times due to the strong rare kicks from the secondary flickering trap. These large excursions at 0.25 s, 1.09 s, 2.21 s, 5.29 s and 8.15 s are marked in the video.
